# Women’s Empowerment and Gender-Related Factors Associated with Maternal Tetanus Protection in 39 Low- and Middle-Income Countries

**DOI:** 10.3390/vaccines13060610

**Published:** 2025-06-06

**Authors:** Katherine Kirkby, Luisa Arroyave, Franciele Hellwig, M. Carolina Danovaro-Holliday, Nasir Yusuf, Shirin Heidari, Stephanie Shendale, Aluísio J. D. Barros, Ahmad Reza Hosseinpoor

**Affiliations:** 1 Department of Data and Analytics, World Health Organization, 20 Avenue Appia, 1211 Geneva, Switzerland; 2 International Center for Equity in Health, Federal University of Pelotas, Rua Mal Deodoro 1160, Pelotas 96020-220, Brazil; 3 Department of Immunization, Vaccines, and Biologicals, World Health Organization, 20 Avenue Appia, 1211 Geneva, Switzerland

**Keywords:** immunization, vaccination, maternal tetanus, gender, gender barriers, empowerment, inequality

## Abstract

**Background:** Tetanus is a vaccine-preventable disease, and therefore vaccination of women of reproductive age or during pregnancy is recommended alongside childhood tetanus vaccination. Gender-related factors related to social empowerment have been established as determinants of health service utilization; however, these social determinants have not yet been explored directly with tetanus vaccination. In response, the aim of this study was to assess overall and country-specific gender-related barriers to maternal tetanus vaccine coverage. **Methods:** We used data from Demographic and Health Surveys (DHS) in 39 countries implemented between 2013 and 2022. Women’s empowerment was measured through three domains of the Survey-based Women’s emPowERment index (SWPER), as well as other gender-related variables. To assess the association between measures of women’s empowerment and gender-related factors and maternal tetanus immunization coverage, we used multilevel logistic models with pooled data from the 39 countries to analyze overall patterns, and we used multivariable logistic regression for each country-specific dataset to analyze country-level associations. **Results:** There were notable variations in the factors associated with tetanus vaccination across countries. Overall, we observed that higher levels of women’s empowerment, as measured through social independence and decision-making autonomy using the SWPER index, were associated with higher odds of maternal tetanus protection, with adjusted odds ratios of 1.23 (95%CI: 1.10–1.37) and 1.20 (95%CI: 1.02–1.40), respectively. However, women’s empowerment related to attitude to violence was not. Higher household wealth was also associated with higher odds of maternal tetanus protection overall. **Conclusions:** Women’s empowerment can improve the uptake of maternal tetanus vaccine. Addressing gender-related barriers may enhance vaccination coverage and contribute to the elimination of maternal and neonatal tetanus as a public health problem. However, these barriers vary from country to country, necessitating country-specific investigations to formulate tailored recommendations.

## 1. Introduction

Tetanus is caused by a neurotoxin produced by the spore-forming bacterium *Clostridium tetani*, which enters the body through a wound or incision [1]. Tetanus is a severe disease, manifesting through muscle spasms, seizures, fever and often death. Case fatality rates range across different settings but are close to 100% without treatment [2]. Encouragingly, tetanus is preventable through vaccination with tetanus toxoid-containing vaccines (TTCVs), which are recommended through a primary series in infancy and a booster series throughout childhood and adolescence.

Tetanus can occur at any stage of life; however, newborns and their mothers are particularly susceptible. Maternal tetanus may occur after procedures related to abortion, miscarriages or non-sterile delivery practices, and neonatal tetanus usually occurs through the infection of the umbilical stump after delivery [1]. Maternal immunization for tetanus protects both mothers and infants since maternal antibodies are transferred to the foetus. Therefore, the vaccination of women of reproductive age or during pregnancy is recommended alongside childhood and booster tetanus vaccination.

Maternal and neonatal tetanus is rare in high-income countries but remains a challenge in low- and some middle-income countries [1]. There are ten countries globally that are yet to eliminate maternal and neonatal tetanus as a public health problem, defined as less than one case per 1000 live births in every district (Afghanistan, Angola, Central African Republic, Nigeria, Pakistan, Papua New Guinea, Somalia, South Sudan, Sudan and Yemen). Despite progress in maternal and neonatal tetanus elimination (MNTE), given the widespread existence of *Clostridium tetani* in the environment globally, vaccination is needed in all countries to both achieve and maintain elimination status.

The extent of tetanus vaccination coverage has been associated with health systems, with low coverage linked with barriers to service delivery, barriers to logistics and barriers to health system management due to inadequate financial and human resources [3]. Tetanus vaccination coverage has also been associated with better antenatal care, given the focus on vaccinations among pregnant women as well as the safety precautions during clinical delivery [3,4,5,6]. Household wealth has also been positively associated with tetanus vaccination coverage, due to better access to health care services, ability to afford transportation and health care costs and higher levels of health literacy and education [7,8,9]. However, beyond the structural and logistical challenges in delivering maternal tetanus vaccination, to better understand inequalities in maternal tetanus vaccination, it is essential to consider sociocultural dimensions, particularly those related to gender norms, roles and relations. These factors can significantly impact and limit women’s empowerment and their access to and utilization of health services.

Women’s empowerment is often measured through constructs like agency, choice, opportunities, resources and power. Empowerment has been described as a dynamic process through which those who have been denied the ability to make choices gain that ability. The process involves interconnected dimensions of resources, agency and achievements [10]. Gender norms, roles and societal gender relations influence the extent of empowerment, making it highly dependent on cultural and contextual factors. Women’s empowerment has been associated with access to and uptake of several health services, including contraceptives and antenatal care, as well as child health interventions such as improved childhood immunization outcomes [11,12,13,14,15,16]. Although gender-related factors associated with social empowerment have been established as determinants of health care service utilization, these social determinants have not yet been explored directly with tetanus vaccination. In response, our objective is to assess gender-related factors associated with maternal tetanus vaccination coverage. We aim to assess this globally, to assess country-specific gender-related barriers to maternal tetanus vaccination and to estimate the compounded advantage of tetanus vaccination among wealthy and empowered women.

## 2. Methods

### 2.1. Data

We utilized data from household surveys conducted by the Demographic and Health Surveys (DHS) Program. These nationally representative surveys involve standardized interviews with women aged 15–49 years, with their detailed methodologies documented in other publications [17]. The sample for this analysis was restricted to ever-married women due to the exposure indicators of interest.

The outcome of interest was maternal tetanus protection, which was defined as the percentage of women with a live birth in the two years preceding the survey whose most recent live birth was protected against tetanus due to receiving a specific number of tetanus toxoid injections during or before their last pregnancy. The indicator calculation is specified within the Guide to DHS Statistics and is aligned with WHO vaccination policy recommendations [18,19].

The primary dimension of interest was women’s empowerment was measured through the Survey-based Women’s emPowERment index (SWPER), which has three domains: (1) social independence, (2) decision-making and (3) attitude to violence [20]. Each domain is composed from several variables collected in the DHS. Social independence includes educational attainment, age at pivotal life events, spousal asset differentials and access to information, all of which support women in achieving their goals. Decision-making reflects the degree to which women are involved in household decisions (including household purchases, health care and visiting relatives). The attitude to violence domain indicates women’s incorporation of gender norms-related acceptability of intimate partner violence (IPV). Higher empowerment in this domain reflects the woman’s rejection that IPV is justified in situations including wife going out without telling the husband, wife neglecting the children, wife arguing with the husband, wife refusing to have sex with the husband and wife burning food. Each SWPER domain has a distinct index value, with 0 representing the global average, while positive values represent greater empowerment and negative values represent lesser empowerment. Based on the distribution of scores, each SWPER domain is classified into three levels of empowerment: low, medium and high [20]. For this paper, we used these SWPER empowerment levels.

Additional variables of interest were selected based on the conceptualization of gender-related and sociodemographic factors that may influence maternal tetanus vaccination coverage, following a literature review. Individual-level demographic and gender-related variables sourced from the DHS data included access to media, pregnancy intentions, employment, ownership of a mobile phone, ownership of a bank account, and autonomy in sexual and reproductive decision-making, including fertility control and exposure to IPV. Country-level gender-related variables included the Gender Development Index (GDI) [21] and ratio of male/female schooling (years) [22]. Sociodemographic factors included age, household wealth and urban/rural place of residence at the individual-level using DHS data. At the country-level these included the gross domestic product (GDP), Gini coefficient, the proportion of population living in urban areas, the proportion of urban population living in slums, and informal settlements or inadequate housing, all sourced from the World Bank DataBank [23]. All country-level data were for the median year of 2019. In addition, the country MNTE status at the time of the survey was considered.

### 2.2. Country Selection

The analysis included 39 low-, lower-middle- and upper-middle-income countries. Countries were considered for inclusion based on the availability of data for maternal tetanus protection, measures of women’s empowerment and the exposure variables of interest from the latest DHS conducted between 2013 and 2022.

### 2.3. Statistical Approach

To assess pooled gender-related barriers, we combined data from the 39 countries. We first assessed bivariate (unadjusted) associations between maternal tetanus protection and each of the individual- and country-level variables of interest. We then analyzed two sets of adjusted models using multilevel logistic models and pooled data across all countries: Set 1 included the three SWPER domains (but not their individual components) and Set 2 included the SWPER composing variables. Set 2 analyses were conducted to investigate whether individual variables within the SWPER index were associated with maternal tetanus protection, irrespective of the overall index domain’s association. The adjusted models included all the individual- and country-level variables of interest, except those omitted by the model due to collinearity.

To assess country-level gender-related barriers to maternal tetanus protection, we analyzed each country’s data separately. We first assessed the bivariate (unadjusted) associations between maternal tetanus protection and our variables of interest using logistic regression. We then assessed adjusted associations using multivariable logistic regression. Like the pooled analysis, we analyzed two sets of adjusted models: Set 1 included the three SWPER domains and Set 2 included the SWPER composing variables.

Analyses considered the survey design (clustering and sampling weights) and population size in the median year (2019), as per the World Population Prospects 2024 Revision. Results are reported as odds ratios, reflecting the chance of maternal tetanus protection compared to the reference group; odds ratios greater than 1 indicate higher odds of protection, while odds ratios less than 1 indicate lower odds of protection. The statistical significance of associations was measured as a *p*-value less than or equal to 0.05.

We investigated whether there were any trends in country-level associations across World Bank income groupings as well as World Health Organization regions. We also assessed the compounded advantage of wealth and gender empowerment in relation to maternal tetanus protection in each country because household wealth encompasses various factors including access to health services and higher levels of health literacy and education. Compound advantage was calculated by multiplying the adjusted effect estimates of wealth and the three SWPER domains. Adjusted associations that were non-significant were reassigned the odds ratio of 1 for the calculation and therefore did not contribute to the final estimate.

### 2.4. Ethical Approval

Ethical approval for DHS data collection was obtained by the national institutions that carried out the surveys, and all analyzed datasets were anonymized and publicly available.

## 3. Results

Of the 39 countries included in this study, 13 were low-income, 19 were lower-middle-income and seven were upper-middle-income countries based on the latest World Bank classification of 2024 (Table 1). All but three countries included in this study (Angola, Papua New Guinea and Pakistan) had eliminated maternal and neonatal tetanus. The prevalence of protection against maternal and neonatal tetanus according to the DHS data ranged from 27.6% (95%CI 25.8–29.5) in Jordan to 91.8% (95%CI 91.6–92.0) in India.

### 3.1. Pooled Assessment of Gender-Related Barriers to Maternal Tetanus Protection

**Figure 1:**
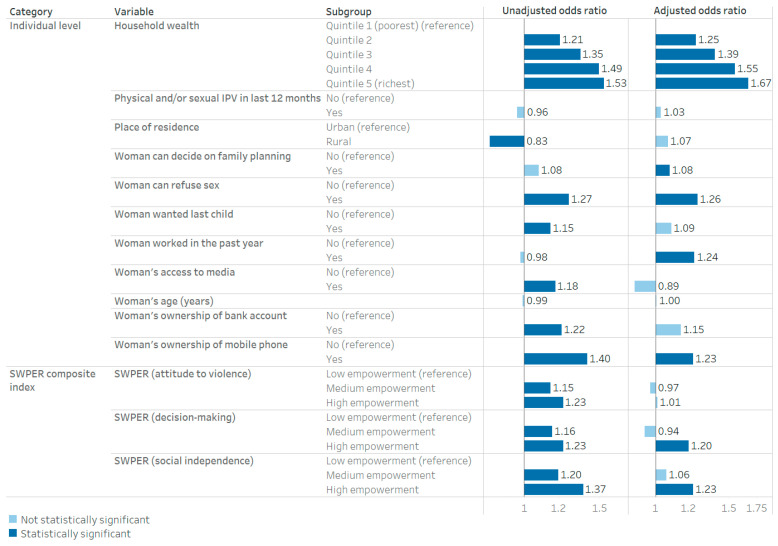
Unadjusted and adjusted associations between maternal tetanus protection and background characteristics across 39 pooled DHS, using a model considering the SWPER domains. Notes: IPV: Intimate partner violence. Unadjusted odds ratios show the association between each variable and maternal tetanus protection without accounting for the other variables. Adjusted odds ratios show the association between each variable and maternal tetanus protection, controlling for all other variables in the figure as well as all country-level variables detailed in Section 2.3. Country-level variables are excluded from the figure since none were statistically significant.

The following presents results from the Set 1 model that considers the SWPER domains; results from the Set 2 model that considers the SWPER composing variables are available in Supplemental File S1 (Table S1). When pooled across all countries, high social independence using the SWPER index was associated with higher maternal tetanus protection compared with low social independence (aOR: 1.23; 95%CI: 1.10–1.37) after adjusting for other variables and controlling for country-level effects (Figure 1). Greater decision-making power using the SWPER index was associated with higher maternal tetanus protection compared with low decision-making power (aOR: 1.20; 95%CI: 1.02–1.40). Attitude to violence as measured by the SWPER index was not associated with maternal tetanus protection.

In addition, in the adjusted model, maternal tetanus protection was positively associated with women being able to decide on fertility control measures (aOR: 1.08; 95%CI: 1.03–1.14); women being able to refuse sex (aOR: 1.26; 96%CI: 1.15–1.38); women working in the past year (aOR: 1.24; 95%CI: 1.12–1.36) and women’s ownership of a mobile phone (aOR: 1.23; 95%CI: 1.12–1.36). Household wealth was associated with maternal tetanus coverage, with increasing magnitude corresponding with higher household wealth when compared to the poorest quintile (aOR for quintile 5: 1.67; 95%CI: 1.31–2.14). After controlling for all variables of interest, no significant associations were observed for women’s age, place of residence, exposure to IPV, pregnancy intentions (wanted last child), access to media and ownership of a bank account. Country-level factors including the GDP, Gini coefficient, GDI, the ratio of male/female schooling and the proportion of population living in various residential settings were also included in the adjusted multilevel model, but none of these had statistically significant associations with maternal tetanus protection.

### 3.2. Country-Specific Assessment of Gender-Related Barriers to Maternal Tetanus Protection

The following presents results from the Set 1 adjusted model, which incorporates the SWPER domains. All country-level results from the Set 1 and Set 2 models are available in an interactive dashboard (https://public.tableau.com/app/profile/who.inequality.monitor/viz/Unadjustedandadjustedassociationsbetweenmaternaltetanuscoverageandbackgroundcharacteristics/Interactivedashboard (accessed on 5 April 2025) and Supplemental File S1 (Tables S2 and S3)).

**Figure 2:**
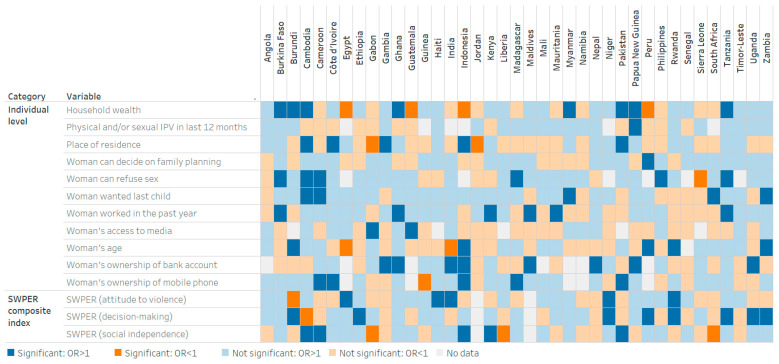
Statistical significance of adjusted associations between maternal tetanus protection and background characteristics in 39 countries, using a model considering the SWPER domains. Notes: OR: Odds ratio, IPV: Intimate partner violence. For variables with multiple subgroups (household wealth and women’s age), the figure shows the adjusted association for the richest quintile and the 35–49 years subgroup.

In 62% of countries (24 out of 39), higher women’s empowerment related to decision-making was positively associated with maternal tetanus protection, with statistical significance observed in eight countries (Burundi, Ethiopia, Niger, Peru, Rwanda, Uganda, Tanzania and Zambia) (Figure 2 and Table 2). In 59% of countries (23 out of 39), higher social independence among women was associated with higher maternal tetanus protection, with statistically significant associations in five countries (Cambodia, Cameroon, Indonesia, Kenya and Pakistan). A negative attitude to violence among women was positively associated with maternal tetanus protection in 56% of countries (22 out of 39), with statistical significance in five countries (Egypt, Haiti, India, Niger and Rwanda).

Factors including women’s decision-making powers regarding contraceptive use and refusal of sex, pregnancy intentions and employment in the past year were positively associated with maternal tetanus protection in over 60% of countries, although statistical significance varied from one to eight countries depending on the variable. Household wealth was positively associated with higher maternal tetanus protection in 64% of countries (25 out of 39), with significant associations in eight countries (Burkina Faso, Burundi, Cambodia, Ghana, Myanmar, Pakistan, Papua New Guinea and Tanzania). Conversely, in four countries (Egypt, Guatemala, Indonesia and Peru), higher household wealth was associated with lower maternal tetanus protection.

Important heterogeneity was observed between countries regarding gender-related and sociodemographic factors associated with maternal tetanus protection. For example, in Burundi, higher household wealth, older age and decision-making empowerment were positively associated with tetanus protection, whereas the attitude to violence domain had a negative association. In Cambodia, higher household wealth, urban residence, women’s ability to refuse sex, pregnancy intentions and social independence were positively associated with maternal tetanus protection, while decision-making power was not. In Angola, Guinea, Jordan, Liberia, Mali, Namibia, Senegal, Sierra Leone and Timor-Leste, none of the variables included in the Set 1 model had a statistically significant positive association with maternal tetanus protection.

Countries with significant associations in the Set 1 model using the SWPER domains did not necessarily have significant associations in the Set 2 model using the SWPER composing variables. For instance, in Burundi women’s negative attitude to violence was found to be associated with lower odds of maternal tetanus protection, but only one of the six composing variables (IPV was considered justified if wife refuses sex) was statistically significant. In Cambodia, women’s decision-making power was associated with lower odds of maternal tetanus protection, yet individual components of this domain showed no statistically significant association with maternal tetanus protection.

Heterogeneity was observed across World Bank income groups and regions (Supplemental File S1, Tables S4 and S5); however, it was difficult to draw robust conclusions due to the small number of countries in some areas. Across low-income countries, the SWPER decision-making domain emerged as having the most positive statistically significant associations (45%, five out of 11 countries). Across lower-middle-income countries, household wealth (29%, six out of 21 countries) was the most common positively associated variable.

### 3.3. Compounded Advantage

In some countries, wealth and gender empowerment compound to increase the likelihood of maternal tetanus protection. Women in the wealthiest quintile of households who also had high measures of empowerment, measured by the social independence, decision-making and attitude to violence SWPER domains, were more likely to have maternal tetanus protection in 18 countries. This was the highest in Pakistan, where women who were both wealthy and had high empowerment had 36 times higher odds of tetanus vaccination than women who were from the poorest households and who had low empowerment measures. In Niger, Rwanda and the United Republic of Tanzania, women had over four times higher odds of being protected, and in a further nine countries (Burkina Faso, Burundi, Cameroon, Ethiopia, Ghana, Haiti, Myanmar, Papua New Guinea and Zambia) the odds were more than double.

## 4. Discussion

The goal of this study was to assess overall and country-specific gender-related barriers to maternal tetanus vaccination and thereby vaccine-induced protection, using the three domains of the SWPER index to measure women’s empowerment. Using pooled data from 39 low- and middle-income countries, we observed that women’s empowerment as measured through autonomy in decision-making and social independence was associated with higher odds of maternal tetanus protection. This reflects findings from other studies of gender barriers to immunization coverage [24]. However, women’s empowerment related to negative attitudes to violence was not associated with maternal tetanus protection. Higher household wealth was also associated with higher maternal tetanus protection using pooled data, as were women’s autonomy to decide on use of contraceptives, women’s ability to refuse sex, women working in the past year and women’s ownership of a mobile phone.

The combination of higher wealth and women’s empowerment appears to increase the magnitude in which women were vaccinated against tetanus in some countries. For example, in 12 countries, women of the highest wealth quintile with high empowerment across the social independence, decision-making and attitudes to violence domains had odds of being vaccinated against tetanus that were more than twice those in women of lower wealth quintiles with low empowerment. This demonstrates that the ways in which gender can interact with other factors such as wealth to shape inequality and mediate immunization outcomes are complex and context specific.

At a country-level the results were varied but, overall, across the 39 countries there were more countries with positive associations (both significant and non-significant) between the gender-related variables and maternal tetanus protection. Higher empowerment related to decision-making, social independence and attitude to violence were associated with maternal tetanus protection in 62%, 59% and 56% of countries, respectively, but these associations were statistically significant in only a few countries. Small sample sizes in some countries and large numbers of variables in the models can lead to reduced statistical power, making it difficult to detect significant associations. Moreover, results were not consistent across countries—for instance, in Burundi, higher decision-making power was associated with higher odds of protection, but higher empowerment in negative attitudes to violence was associated with lower odds of protection. In Ethiopia, only higher decision-making power was associated with higher odds of protection. Associations therefore vary from country to country, necessitating investigation of country-specific results and further research to formulate tailored recommendations that target the different pathways through which social independence and decision-making can influence immunization outcomes. This reflects the diversity of women’s experiences across households, communities and countries, and the varied ways that gender intersects with other dimensions and experiences of exclusion [25]. Women’s empowerment as measured through their negative attitudes towards violence was not associated with maternal tetanus protection in the pooled analysis. This may be due to the variation in these associations across countries, as well as the role of the IPV attitudes variables that are within the SWPER composite index. For example, higher empowerment measured by negative attitude to violence was associated with higher odds of maternal tetanus protection in five countries, lower odds of maternal tetanus protection in one country and no significant association in 31 countries. When we assessed women’s empowerment by the composing variables that make up the SWPER index instead of the domains, varying results were observed related to attitude to violence within the same countries. For example, in both Guinea and Nepal, acceptance of IPV due going out without telling a husband was positively associated with maternal tetanus protection, but arguing with a husband was negatively associated. In Burkina Faso, attitudes reflecting acceptance of IPV due to neglecting children and refusing sex with husband were positively associated with maternal tetanus protection, but IPV justification due to arguing with a husband or burning food were negatively associated. These variations may be due to misogyny and gender-based violence being complex, multifaceted and often driven by cultural norms related to gender roles, which vary across settings and may not directly impact vaccination decisions. Moreover, they may highlight a potential limitation of the SWPER index in capturing these complexities.

Our research suggests a link between tetanus protection and women’s social independence and decision-making power as measured using the SWPER index, at least in some settings, which aligns with previously published studies that identify gender-related barriers such as maternal education and social status as significant obstacles to improving immunization coverage [26,27,28]. Through the SWPER index and additional variables, we included a wide range of gender-related barriers in our analyses including education, access to media and information, power and agency within the home, gender-based violence, decision-making abilities, employment and wealth. Yet, other important barriers that were not captured in the analysis include health literacy, mobility and transportation, time constraints, lack of female health workers, long wait times at health care centers, religious beliefs and cultural practices [29]. The SWPER index has limitations in that it does not capture some important measures of empowerment, only applies to women who are married or in a union and can only be calculated for countries with DHS data availability for the relevant variables [20].

Women’s empowerment is multidimensional, complex and context specific and therefore challenging to measure. This may contribute to the variation in results across countries. Sex and gender may drive tetanus protection in several distinct ways across populations. For instance, sociocultural norms and gender roles can affect women’s access to health care services, including tetanus vaccination, as women may face restrictions on mobility or decision-making power within households [7]. Additionally, inequalities in education and economic opportunities between genders can lead to differences in health literacy and financial ability to seek vaccination [30]. Adopting gender-transformative strategies that challenge harmful gender norms, address structural gender inequalities in health care and consider the broader gender dynamics influencing health decisions is key for enhancing equity in immunization [31]. Such approaches align with recommendations from key global immunization stakeholders, including the World Health Organization’s Immunization Agenda 2030 and Gavi 5.0 [32,33].

There are several limitations that should be considered when interpreting these results. First, the data for this study was limited to 39 low- and middle-income countries with a DHS dataset; findings from other settings and populations may differ. Second, it is important to note that no definitive conclusions about causality can be drawn from the findings of this cross-sectional study, and such interpretation should be avoided unless more direct studies are conducted. Third, small sample sizes in country-specific analyses and the large number of associations tested within the model can potentially render results statistically insignificant and increase the likelihood of spurious associations. We tested the statistical significance of our findings to determine if the observed relationship is unlikely to have occurred by chance. While many of the 95% confidence intervals were relatively narrow—indicating precise estimates—some were wider, particularly in countries with smaller sample sizes or more variability in the data. Fourth, the data may not reflect the prevailing situations in these countries, given that the surveys may have been conducted several years ago. Fifth, DHS data are collected through interviewer-administered surveys, which consists of self-reported experiences, history and health. Self-reported measures may be subject to recall bias or social desirability bias. Sixth, we did not adjust for health care access and availability of vaccination services, as these variables are not available in DHS datasets. The seventh, and probably most important limitation, is that the study may be subject to misclassification of the outcome of maternal tetanus protection. Usually, health workers ask pregnant women about the number of the doses received during the pregnancy of the most recent birth and the number of doses received during previous pregnancies, including those through mass vaccination campaigns and outreach sessions without the sort of precision outlined in the DHS methods. It may be difficult for women to remember all vaccines received and for DHS interviewers to collect data from respondents in such a systematic manner. Eighth, our analysis only reflects the association between women’s empowerment and maternal tetanus protection, and the results cannot be assumed to relate to other vaccinations during pregnancy.

Finally, when considering the ultimate goal of protection at birth against tetanus, clean delivery and umbilical cord practices play an important role, in addition to tetanus vaccination [34]. The apparent lack of association between maternal tetanus vaccination coverage and MNTE (for example, coverage is low in South Africa and Jordan, despite both countries achieving MNTE) is explained by the fact that countries can attain MNTE through access to clean delivery.

## 5. Conclusions

Our study across 39 low- and middle-income countries suggests that women’s social empowerment and decision-making power are associated with maternal tetanus protection. However, these associations varied widely across countries. Therefore, gender-related barriers may hinder the uptake maternal tetanus vaccine, at least in some settings, and addressing these barriers may enhance vaccination coverage and contribute to the elimination of maternal and neonatal tetanus as a public health problem. However, gender-related barriers and the pathways through which gender influences maternal tetanus protection vary from country to country, necessitating investigation of country-specific results to formulate tailored recommendations.

## Figures and Tables

**Table 1:** Status of protection against maternal and neonatal tetanus, 39 countries (DHS 2013–2022).

Country	DHS Survey Year	World Bank Income Group ^a^	Protection Against Maternal and Neonatal Tetanus			Elimination Status ^b^
			Prevalence (%)	95%CI Lower Limit	95%CI Upper Limit	
India	2019	Lower-middle-income	91.8	91.6	92.0	Eliminated
Senegal	2019	Lower-middle-income	87.1	85.4	88.7	Eliminated
Sierra Leone	2019	Low-income	85.1	83.7	86.5	Eliminated
Burundi	2016	Low-income	84.3	83.1	85.4	Eliminated
Liberia	2019	Low-income	82.7	80.5	84.7	Eliminated
Uganda	2016	Low-income	80.6	79.4	81.7	Eliminated
Rwanda	2019	Low-income	79.1	77.8	80.4	Eliminated
Zambia	2018	Lower-middle-income	78.6	77.2	79.9	Eliminated
Guatemala	2014	Lower-middle-income	77.9	76.6	79.1	Eliminated
Haiti	2016	Low-income	74.6	72.6	76.4	Eliminated
Egypt	2014	Lower-middle-income	74.3	73.0	75.6	Eliminated
Madagascar	2021	Low-income	73.1	71.4	74.7	Eliminated
Myanmar	2015	Lower-middle-income	71.9	69.2	74.4	Eliminated
Timor Leste	2016	Lower-middle-income	71.5	69.4	73.5	Eliminated
Cameroon	2018	Lower-middle-income	71.0	69.0	72.9	Eliminated
Gambia	2019	Low-income	70.6	68.7	72.4	Eliminated
Maldives	2016	Upper-middle-income	69.2	66.6	71.7	Eliminated
Gabon	2019	Upper-middle-income	69.2	65.9	72.4	Eliminated
Pakistan	2017	Lower-middle-income	68.9	66.2	71.4	Endemic
Peru	2022	Upper-middle-income	67.5	66.6	68.5	Eliminated
Angola	2015	Upper-middle-income	65.9	64.1	67.7	Endemic
Namibia	2013	Upper-middle-income	65.8	63.6	68.0	Eliminated
Tanzania	2022	Lower-middle-income	64.1	62.7	65.5	Eliminated
Nepal	2022	Lower-middle-income	61.9	60.0	63.9	Eliminated
Cambodia	2021	Lower-middle-income	61.8	60.2	63.3	Eliminated
Guinea	2018	Low-income	59.1	56.4	61.7	Eliminated
Indonesia	2017	Lower-middle-income	57.5	56.3	58.8	Eliminated
Niger	2021	Low-income	55.1	51.7	58.5	Eliminated
Ghana	2022	Lower-middle-income	54.5	52.5	56.4	Eliminated
Kenya	2022	Lower-middle-income	52.3	51.1	53.5	Eliminated
Cote d’Ivoire	2021	Lower-middle-income	50.8	49.3	52.4	Eliminated
Philippines	2022	Lower-middle-income	50.0	48.2	51.8	Eliminated
Mali	2018	Low-income	49.6	47.1	52.0	Eliminated
Ethiopia	2016	Low-income	48.9	46.2	51.7	Eliminated
Mauritania	2019	Lower-middle-income	48.2	46.1	50.2	Eliminated
Burkina Faso	2021	Low-income	47.4	45.8	49.0	Eliminated
Papua New Guinea	2016	Lower-middle-income	37.9	35.3	40.6	Endemic
South Africa	2016	Upper-middle-income	35.1	32.5	37.8	Eliminated
Jordan	2017	Upper-middle-income	27.6	25.8	29.5	Eliminated

^a^ Based on 2024 World Bank classification. ^b^ Defined as less than one case per 1000 live births in every district in the country. 95%CI: 95% confidence interval.

**Table 2:** Count of the number of countries by type of adjusted association between maternal tetanus protection and background characteristics, using a model considering the SWPER domains.

Category	Variable	Significant: OR > 1	Significant: OR < 1	Not Significant: OR > 1	Not Significant: OR < 1	No Data
Individual level	Household wealth	8	4	17	10	
	Physical and/or sexual IPV in last 12 months	1		20	12	6
	Place of residence	5	2	14	18	
	Woman can decide on family planning	1		24	14	
	Woman can refuse sex	6	1	20	6	6
	Woman wanted last child	5		24	10	
	Woman worked in the past year	6		19	14	
	Woman’s access to media	2		12	20	5
	Woman’s age	5	2	14	17	1
	Woman’s ownership of bank account	8		10	14	7
	Woman’s ownership of mobile phone	5	1	20	8	5
SWPER composite index	SWPER (attitude to violence)	5	1	17	14	2
	SWPER (decision-making)	8	1	16	12	2
	SWPER (social independence)	5	3	18	11	2

Notes: OR: Odds ratio, IPV: Intimate partner violence. For variables with multiple subgroups (household wealth and women’s age), the table reflects the adjusted association for the richest quintile and the 35–49 years subgroup.

## Data Availability

All analyses were carried out using publicly available datasets that could be obtained directly from the DHS Program (dhsprogram.com).

## Supplementary Materials

The following supporting information can be downloaded at: https://www.mdpi.com/article/10.3390/vaccines13060610/s1, Supplementary File S1, Table S1: Unadjusted and adjusted associations between maternal tetanus protection and background characteristics across 39 pooled DHS; Table S2: Unadjusted and adjusted associations between maternal tetanus protection and background characteristics in 39 countries, using a model considering the SWPER index domains (Set 1); Table S3: Unadjusted and adjusted associations between maternal tetanus protection and background characteristics in 39 countries, using a model considering the SWPER composing variables (Set 2); Table S4: Count of the number of countries by World Bank income group and type of adjusted association between maternal tetanus protection and background characteristics, using a model considering the SWPER index domains; Table S5: Count of the number of countries by WHO region and type of adjusted association between maternal tetanus protection and background characteristics, using a model considering the SWPER index domains. Country-level unadjusted and adjusted associations using both models (including the three SWPER domains and including the SWPER composing variables) are available in an interactive dashboard (https://public.tableau.com/app/profile/who.inequality.monitor/viz/Unadjustedandadjustedassociationsbetweenmaternaltetanuscoverageandbackgroundcharacteristics/Interactivedashboard).
